# Procyanidins A1 and B1 Suppress PEDV CV777 by Modulating Mitophagy

**DOI:** 10.3390/v18070758

**Published:** 2026-07-10

**Authors:** Yujing Weng, Jialin Li, Cong Ma, Jiufeng Wang, Xiaojia Wang

**Affiliations:** 1 National Key Laboratory of Veterinary Public Health and Safety, College of Veterinary Medicine, China Agricultural University, Beijing 100193, China; 13509377604@163.com (Y.W.); lijialin_scau@163.com (J.L.); 2 Sanya Institute, China Agricultural University, Sanya 572000, China; 3 Health Management Institute, The Second Medical Center & National Clinical Research Center for Geriatric Diseases, Chinese PLA General Hospital, Beijing 100853, China

**Keywords:** porcine epidemic diarrhea virus, procyanidin, mitophagy, molecular docking

## Abstract

Porcine epidemic diarrhea virus (PEDV) causes severe enteric disease and high mortality in piglets, yet effective therapeutic options remain scarce. To identify novel antivirals, we evaluated the inhibitory potential and molecular mechanisms of four procyanidin subtypes (A1, A2, B1, and B2) in Vero cells. Our results indicate that procyanidins A1 and B1 possess superior anti-PEDV activity compared with their analogs, acting primarily through direct virucidal inactivation (*p* < 0.001) and by blocking viral adsorption (*p* < 0.001), internalization (*p* < 0.001), and replication (*p* < 0.05). The SI of procyanidin A1 and B1 in Vero cells are 22.4 and 10.8. At the cellular level, mitochondrial membrane dynamics proteins regulate mitophagy-related proteins. PEDV infection disrupts mitochondrial dynamics and hijacks the autophagic machinery, which is characterized by the upregulation of the fission protein DRP1 and the mitophagy regulator Parkin, concurrent with the decrease of p62 and the increase in LC3-II. Treatment with procyanidins A1 and B1 effectively counteracted these alterations, restoring p62 levels (*p* < 0.05), decreasing LC3-II levels (*p* < 0.001), reducing autolysosome formation, and reversing the aberrant upregulation of DRP1 (*p* < 0.001) and Parkin (*p* < 0.05) to maintain mitochondrial homeostasis. Molecular docking results suggested that the potential binding affinity between procyanidin A1 and the mitochondrial protein Parkin was significantly higher than that of other configurations. Structural analysis further indicated that the presence of an additional ether bond and the trans configuration of the terminal catechin unit in procyanidin A1 might be important factors contributing to its superior antiviral efficacy. These results suggest that procyanidins may inhibit PEDV by inactivating viral particles or blocking their adsorption and internalization to prevent viral entry, while simultaneously modulating host mitochondrial proteins to suppress the replication of viruses that have already entered the cells, thereby supporting the potential of procyanidins A1 and B1 as effective antiviral candidates.

## 1. Introduction

Porcine epidemic diarrhea virus (PEDV), a member of the Coronaviridae family, causes severe vomiting, diarrhea, dehydration, and death in newborn piglets. In the winter of 2010, a novel highly pathogenic GII PEDV variant emerged in southern China, characterized by morbidity and mortality rates exceeding 80%. In recent years, this high-pathogenicity GII variant has spread globally, causing massive economic losses to the swine industry. Statistics indicate that in 2013, the morbidity rate of piglets infected with pathogenic strains in the United States approached 100%, with mortality rates reaching 90–95% [1]. Similarly, piglet mortality exceeded 70% in Germany in 2014 [2] and ranged from 80% to 95% in Mexico between 2016 and 2018 [3]. Given that currently available commercial vaccines do not offer complete protection against the virus, there is an urgent need to develop effective strategies for the prevention and control of PEDV.

Autophagy is a highly conserved intracellular degradation pathway essential for maintaining cellular homeostasis through the degradation and recycling of damaged organelles and misfolded cytoplasmic proteins. It is typically activated by stress responses such as viral infection or organelle damage [4,5]. Accumulating evidence suggests that autophagy serves as a critical cellular defense mechanism closely linked to the viral life cycle, eliminating virions via lysosomal degradation to restrict viral replication [6]. However, autophagy plays a dual role in the antiviral innate immune response. In the context of PEDV infection, while autophagy can assist cells in recognizing and degrading viral components to inhibit replication, PEDV can conversely hijack the autophagic machinery to facilitate its own replication, assembly, and release [7], representing a complex interplay between the virus and the host.

Mitochondria, which serve as hubs for cellular bioenergetics and biosynthesis, maintain a dynamic balance between fission and fusion, which is vital for cellular homeostasis. Viral infections often disrupt mitochondrial homeostasis, impairing the normal cellular energy supply and metabolism to facilitate viral propagation within host cells [8,9]. Most viruses have evolved mechanisms to hijack host metabolic resources for their replication [10]. Our previous studies revealed that PEDV infection induces excessive mitochondrial fission [11]. This excessive fission subsequently activates mitophagy. Mitophagy is a selective form of autophagy that specifically recognizes and eliminates damaged, depolarized, or dysfunctional mitochondria. Mitophagy maintains normal cellular function by regulating mitochondrial quality. This process may represent a key mechanism employed by PEDV to evade host innate immunity.

Procyanidins (PCs) are plant secondary metabolites and constitute the most abundant subclass of flavonoids found in nature. They are polymeric structures composed of flavan-3-ol units linked by C–C bonds and are commonly found in fruits and vegetables [12]. These monomers are primarily classified into A-type (linked by C_2_-O-C_7_ or C_2_-O-C_5_) and B-type (linked by C_4_-C_8_ or C_4_-C_6_) [13]. Procyanidins possess potent biological activities; in addition to their antioxidant properties, studies have shown that they can inhibit pathogen adhesion [14]. Furthermore, it has been reported that procyanidins exhibit antagonistic activity against coronaviruses, including SARS-CoV-2 and MERS-CoV [15,16]. Our previous research demonstrated that procyanidins significantly inhibit PEDV infection and ameliorate PEDV-induced membrane morphological changes [11]; however, the underlying mechanisms remain unclear. Therefore, this study aimed to investigate whether procyanidins exert their anti-PEDV effects by influencing the mitophagy process. These findings provide a theoretical basis for the potential application of procyanidins as novel and effective anti-PEDV agents.

## 2. Materials and Methods

### 2.1. Reagents and Antibodies

Procyanidin A1 (CAS No. 103883-03-0, purity ≥ 99.96%, HY-N2344, 1 µg/mL = 1.735 µM), procyanidin A2 (CAS No. 41743-41-3, purity ≥ 99.85%, HY-N2343, 1 µg/mL = 1.735 µM), procyanidin B1 (CAS No. 20315-25-7, purity ≥ 99.28%, HY-N0795, 1 µg/mL = 1.729 µM), procyanidin B2 (CAS No. 29106-49-8, purity ≥ 99.79%, HY-N0796, 1 µg/mL = 1.729 µM), and ribavirin (CAS No. 36791-04-5, purity ≥ 99.96%, HY-B0434) were purchased from MedChemExpress (South Brunswick, NJ, USA).

Antibodies against DRP1 (1:1000, Cat#A21968) and MFN1 (1:1000, Cat#A9880) were obtained from ABclonal (Wuhan, China). Antibodies against P62/SQSTM1 (1:5000, Cat#18420-1-AP), β-actin (1:10,000, Cat#20536-1-AP), HRP-conjugated goat anti-mouse IgG (H + L) (1:10,000, Cat#SA00012-1), and HRP-conjugated goat anti-rabbit IgG (H + L) (1:10,000, Cat#SA00001-2) were purchased from Proteintech (Wuhan, China). An anti-PINK1 antibody (1:1000, Cat#ab216144) was purchased from Abcam (Cambridge, UK). Anti-Parkin antibody (1:1000, Cat# F0296) was obtained from Selleckchem (Houston, TX, USA). The anti-LC3A/B antibody (1:1000, Cat#4108) was purchased from Cell Signaling Technology (CST) (Danvers, MA, USA). PEDV-N monoclonal antibody (1:1000 for Western blot, 1:500 for IFA, Cat# SD-1-5) was purchased from Medgene Labs (Brookings, SD, USA).

### 2.2. Cells and Virus

The PEDV CV777 strain (GenBank Accession No. AF353511), Vero cells and IPEC-J2 cells were used in this study. PEDV was propagated and purified in Vero cells. Vero cells and IPEC-J2 cells were cultured in Dulbecco’s modified Eagle’s medium (DMEM) (Cytiva, Shanghai, China) supplemented with 10% fetal bovine serum (FBS) (Gibco, Waltham, MA, USA) and 1% penicillin–streptomycin (Gibco, Waltham, MA, USA). The cells were maintained in a humidified incubator at 37 °C with 5% CO_2_.

### 2.3. Transfection

Plasmids encoding DNM1L (GenBank Accession No. NM_012062.5) and MFN1 (GenBank Accession No. NM_033540.3) were created using pcDNA3.1 vectors containing FLAG tags, and the primers used for plasmid construction are listed in Table 1. The mCherry-GFP-LC3 reporter was purchased from Fenghui (Beijing, China). The siRNAs targeting DRP1 and MFN1 were designed and synthesized by an external siRNA service (Shanghai GenePharma Co., Ltd., Shanghai, China). Vero cells or IPEC-J2 cells were cultured in 24-well plates to 80% confluence. Plasmids were transfected using Lipofectamine™ 3000 Transfection Reagent (Invitrogen, Carlsbad, CA, USA), and siRNAs were transfected using Lipofectamine™ RNAiMAX Transfection Reagent (Invitrogen, Carlsbad, CA, USA). At 24 h post-transfection, the cells were either mock-infected or infected with PEDV (MOI = 0.5) for 48 h. The cells transfected with the mCherry-GFP-LC3 plasmid were fixed with 4% paraformaldehyde, and mCherry-GFP-LC3 fluorescence images were acquired using a fluorescence microscope (Nikon, Tokyo, Japan).

### 2.4. Western Blot

The cell samples were lysed with RIPA lysis buffer on ice and pipetted repeatedly to ensure complete lysis. The lysates were centrifuged at 13,000 rpm for 15 min at 4 °C, and the supernatants were collected. The total protein concentration was normalized via a bicinchoninic acid (BCA) assay. The samples were mixed with 5× loading buffer and denatured by heating at 95 °C for 10 min. The protein samples were separated via SDS–PAGE and transferred onto PVDF membranes. The membranes were blocked with 5% BSA for 1 h at 25 °C, incubated with primary antibodies overnight at 4 °C, and then incubated with secondary antibodies for 1 h at 25 °C. The protein bands were visualized and analyzed via the Tanon 5200 Chemiluminescent Imaging System.

### 2.5. Indirect Immunofluorescence Assay (IFA)

Vero cells were seeded in culture plates and grown to approximately 80% confluence before being subjected to PEDV infection and drug treatment. Following treatment, the cells were fixed with 4% paraformaldehyde (40 µL/well) for 30 min at RT and washed with PBS. The cells were then permeabilized with 0.1% Triton X-100 (10 µL/well) on ice for 1 min and washed with PBS. Blocking was performed via the addition of 5% BSA (40 µL/well) for 1 h at RT, followed by washing with PBS. The cells were incubated with a PEDV-N monoclonal antibody (1:500 dilution, 20 µL/well) overnight at 4 °C. After being washed with PBS, the cells were incubated with HRP-conjugated goat anti-mouse IgG (H + L) (1:10,000 dilution, 20 µL/well) for 1 h at RT and washed with PBS. Nuclei were counterstained with DAPI (30 µL/well) for 2 min at RT in the dark. After a final wash with PBS, the cells were observed and photographed under a fluorescence microscope.

### 2.6. Selectivity Index

Vero or IPEC-J2 cells were seeded into 96-well plates and cultured until reaching approximately 70% confluence. The cells were then incubated in DMEM supplemented with 2% FBS containing serially diluted concentrations of procyanidin A1 (12.5, 25, 50, 75, 100, 150, 200, 300 μg/mL) or procyanidin B1 (25, 50, 100, 150, 200, 300, 400, 500 μg/mL) for 24 h. Cell viability was assessed using a Cell Counting Kit-8 (CCK-8, Solarbio, Beijing, China). Absorbance was measured at 450 nm using a Varioskan™ LUX multimode microplate reader (Thermo Fisher Scientific, Waltham, MA, USA). The 50% cytotoxic concentration (CC_50_) was calculated by curve fitting using GraphPad 9.2.0 software.

Cells seeded in 96-well plates and grown to approximately 70% confluence were infected with PEDV at a multiplicity of infection (MOI) of 0.5 for 2 h. Thereafter, the medium was replaced with 2% DMEM containing graded concentrations of procyanidin A1 (1.25, 2.5, 5, 10, 20, 40 μg/mL) or procyanidin B1 (2.5, 5, 10, 20, 40, 80 μg/mL), and the cells were incubated for an additional 24 h. The antiviral effect was evaluated by immunofluorescence assay (IFA). Fluorescence intensity was measured using the Varioskan™ LUX microplate reader. The 50% effective concentration (EC_50_) was determined by curve fitting with GraphPad software.

The selectivity index was calculated as the ratio of CC_50_ to EC_50_ (SI = CC_50_/EC_50_).

### 2.7. Quantitative Real-Time PCR (RT–qPCR)

Total RNA was extracted from Vero cells using the Total RNA Extraction Kit (Takara, Osaka, Japan) according to the manufacturer’s instructions. RNA concentration and purity were determined using a NanoDrop spectrophotometer (Thermo Fisher Scientific, Waltham, MA, USA), with an A260/A280 ratio between 1.8 and 2.0. Total RNA was reverse-transcribed into cDNA and genomic DNA was removed using a TransScript^®^ All-in-One SuperMix (TransGen Biotech, Beijing, China) in a total volume of 20 µL. The reaction was performed at 42 °C for 15 min, followed by 85 °C for 5 s. qPCR was performed using a PerfectStart^®^ Green qPCR SuperMix (TransGen Biotech, China) on a StepOnePlus™ Real-Time PCR System (Applied Biosystems, Waltham, MA, USA). The total reaction volume was 20 µL. The thermal cycling protocol was: initial denaturation at 94 °C for 30 s, followed by 40–45 cycles of 94 °C for 10 s (denaturation) and 60 °C for 30 s (annealing/extension). The primer sequences for PEDV-N (GenBank accession number NC_003436.1) are listed in Table 2. Absolute quantification was performed via the standard curve method. The absolute copy numbers of PEDV-N in each sample were calculated by interpolating the Cq values onto the respective standard curves.

### 2.8. Molecular Docking

Molecular docking of procyanidin with mitofusin 1 (MFN1), dynamin-related protein 1 (DRP1), PTEN-induced kinase 1 (PINK1), and Parkin was performed via QuickVina-W. The 3D structures of procyanidin A1 (CID: 5089889), B1 (CID: 11250133), A2 (CID: 124025) and B2 (CID: 122738) were retrieved from PubChem (https://pubchem.ncbi.nlm.nih.gov (accessed on 24 December 2025)). The crystal structures of MFN1 (PDB ID: 5GOF), DRP1 (PDB ID: 3W6O), PINK1 (PDB ID: 9EII), and Parkin (PDB ID: 5C1Z) were obtained from the RCSB Protein Data Bank (www.rcsb.org (accessed on 24 December 2025)). Input files for docking were prepared via the QuickVina-W suite. The binding pose with the highest Vina docking score was selected, and visualization and analysis were performed via Discovery Studio (www.3ds.com (accessed on 24 December 2025)).

### 2.9. Statistical Analysis

Statistical analyses were performed using IBM SPSS 26.0 (IBM Corp., Armonk, NY, USA) and were generated using GraphPad Prism 9.2.0 software (GraphPad Software, Boston, MA, USA). Data are presented as mean ± SD and are derived from at least three independent experiments. Comparisons between two groups were made using unpaired *t*-tests. Statistical significance is indicated as follows: * *p* < 0.05, ** *p* < 0.01, and *** *p* < 0.001.

## 3. Results

### 3.1. PEDV Infection Activates Autophagy and Mitophagy in Vero Cells

**Figure 1:**
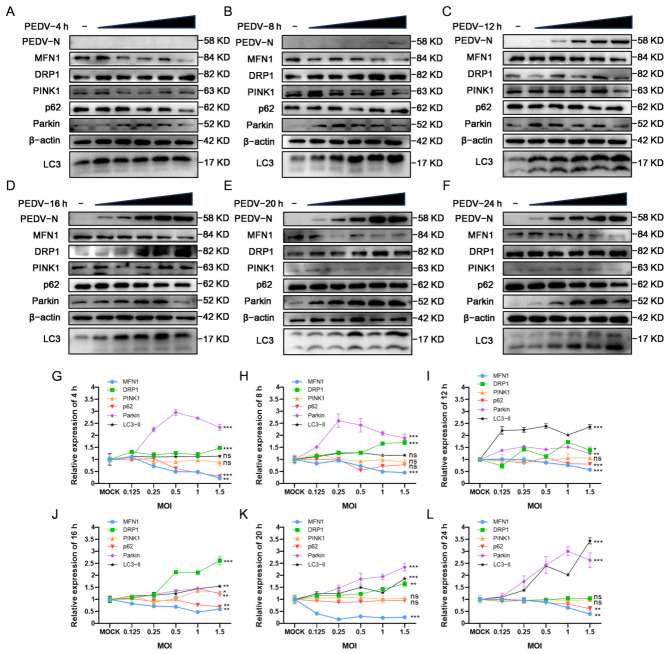
Effects of PEDV infection at gradient MOI on the expression of autophagy, mitochondrial dynamics, and mitophagy-related proteins in Vero cells at different time points (*n* = 3). (**A**–**F**) Vero cells were infected with PEDV at MOI of 0.125, 0.25, 0.5, 1, and 1.5. The cell lysates were harvested at 4 h (**A**), 8 h (**B**), 12 h (**C**), 16 h (**D**), 20 h (**E**), and 24 h (**F**) postinfection, and protein expression was detected via Western blot with the indicated antibodies. (**G**–**L**) Densitometric analysis of protein bands (MOI = 1.5) was performed via ImageJ 1.54g software, corresponding to (**A**–**F**), respectively. ns: *p* > 0.05, *: *p* < 0.05, **: *p* < 0.01, ***: *p* < 0.001.

Viral infections often disrupt cellular metabolic processes, including autophagy. Our previous studies also indicated that PEDV infection affects mitochondrial membrane morphology. To investigate the temporal dynamics of PEDV-induced cellular alterations, cells grown to approximately 80% confluence were infected with PEDV at graded MOIs, then incubated at 37 °C for 4, 8, 12, 16, 20, and 24 h, respectively. After incubation, cells were washed three times with PBS, and total protein was extracted for Western blot analysis (Figure 1A–F). The results were normalized and analyzed using ImageJ 1.54g software (Figure 1G–L). The results revealed that the expression of the PEDV N protein gradually increased after 8 hpi, indicating that viral replication accelerated significantly after this time point. PEDV infection led to increased LC3-II expression and decreased p62 expression. Concurrently, the expression of MFN1 was downregulated, whereas DRP1 expression was upregulated. Given that DRP1 primarily regulates mitochondrial fission, whereas mitochondrial fusion relies mainly on proteins such as MFN1 and OPA1 [17], these results suggest that PEDV infection disrupts mitochondrial dynamics, leading to excessive fission and morphological abnormalities, which is consistent with our previous findings. Furthermore, we observed varying degrees of upregulation of Parkin expression at all time points following PEDV infection, providing further evidence that mitophagy was significantly activated. The increase in LC3-II and the decrease in p62 suggest PEDV induces autophagy, possibly involving enhanced autophagic flux.

### 3.2. The Mitochondrial Membrane Dynamics Proteins DRP1 and MFN1 Regulate the Mitophagy-Related Proteins PINK1 and Parkin

**Figure 2:**
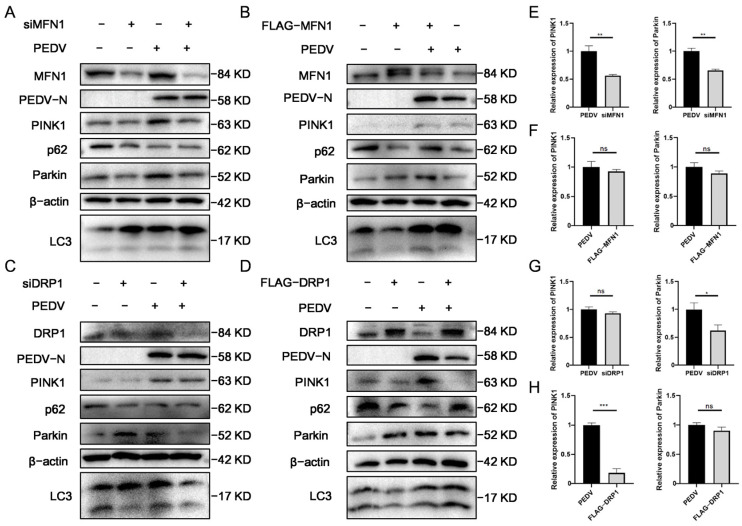
Effects of silencing or overexpressing the mitochondrial membrane dynamics proteins DRP1/MFN1 on the mitophagy proteins PINK1 and Parkin (*n* = 3). (**A**–**D**) Following transfection with siRNA or plasmids, Vero cells were infected with PEDV at MOI of 0.5. (**A**) Transfection with siMFN1. (**B**) Transfection with FLAG-MFN1. (**C**) Transfection with siDRP1. (**D**) Transfection with FLAG-DRP1. (**E**–**H**) Densitometric analysis of protein bands was performed via ImageJ 1.54g software, corresponding to (**A**–**D**), respectively. ns: *p* > 0.05, *: *p* < 0.05, **: *p* < 0.01, ***: *p* < 0.001.

To evaluate the effects of mitochondrial dynamics-related proteins on mitophagy, we silenced or overexpressed DRP1 and MFN1 proteins after PEDV infection via transfection with siRNA and FLAG-tagged plasmids, respectively, and monitored the changes in PINK1 and Parkin protein expression levels (Figure 2A–D). The results demonstrated that silencing MFN1 inhibited the expression of PINK1 and Parkin proteins (Figure 2E), whereas overexpressing MFN1 did not result in any significant changes (Figure 2F). Furthermore, silencing DRP1 led to a decrease in Parkin protein expression (Figure 2G), while overexpressing DRP1 significantly reduced the expression of PINK1 protein (Figure 2H).

### 3.3. Procyanidins A1 and B1 Exhibit Superior Inhibitory Effects Against PEDV

**Figure 3:**
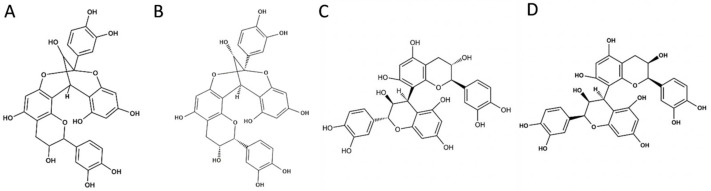
The chemical structures of procyanidins A1, A2, B1, and B2. (**A**) Procyanidin A1. (**B**) Procyanidin A2. (**C**) Procyanidin B1. (**D**) Procyanidin B2.

Procyanidins have different configurations. We studied the main configurations A1, A2, B1, and B2, among which A1 and A2 are isomers of each other, and B1 and B2 are also isomers of each other (Figure 3).

**Figure 4:**
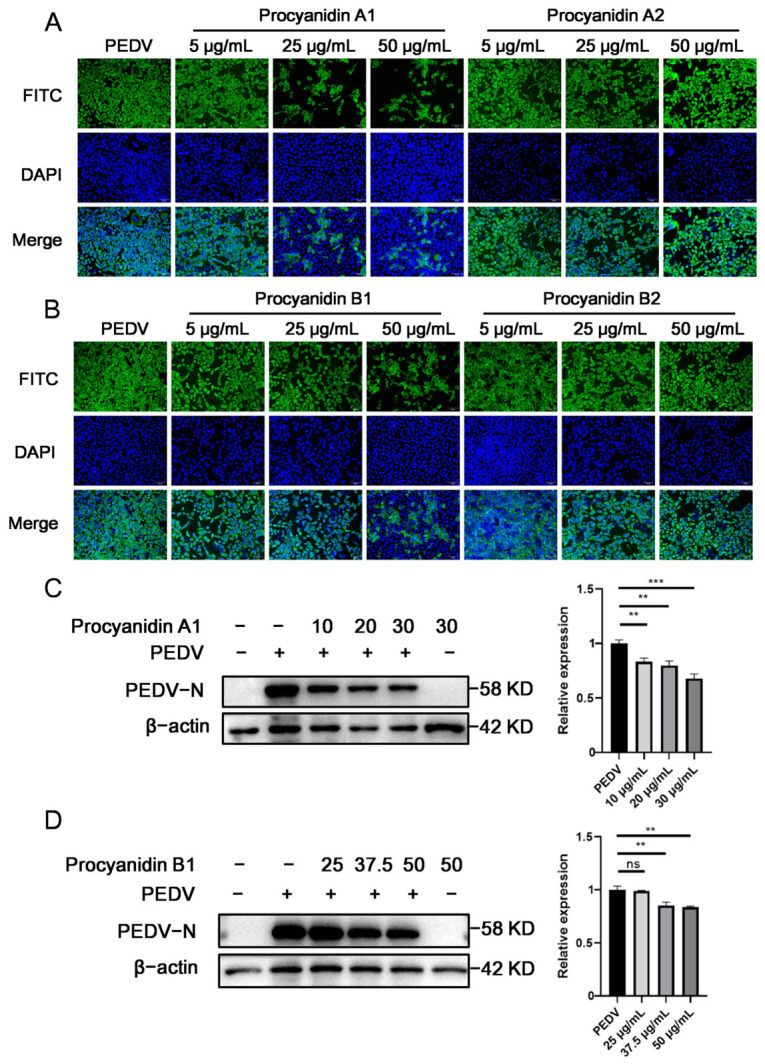
Inhibitory effects of different procyanidin configurations on PEDV infection (*n* = 3). (**A**,**B**) Vero cells were infected with PEDV (MOI = 0.5) for 2 h and treated with the indicated concentrations of procyanidins for 20 h. The cells were fixed, permeabilized, and immunostained for indirect immunofluorescence imaging. PEDV-N: green; DAPI: blue. (**A**) Procyanidin A1 and A2 at 5, 25, and 50 μg/mL. (**B**) Procyanidin B1 and B2 at 5, 25, and 50 μg/mL. (**C**,**D**) Vero cells were infected with PEDV (MOI = 0.5) for 2 h and subsequently treated with the indicated concentrations of procyanidins for 20 h. Total protein was extracted for Western blot analysis. (**C**) Procyanidin A1 at 10, 20 and 30 μg/mL. (**D**) Procyanidin B1 at 25, 37.5, and 50 μg/mL. ns: *p* > 0.05, **: *p* < 0.01, ***: *p* < 0.001.

To evaluate the antiviral efficacy of procyanidins with different structures, we selected 20 h post infection (hpi) as the treatment duration, on the basis of the stable protein expression trends observed in previous time-course experiments. PEDV-infected Vero cells were treated with gradient concentrations of procyanidins A1, A2, B1, and B2. The immunofluorescence assay results (Figure 4A,B) demonstrated that the A1 and B1 configurations exhibited superior inhibitory effects on PEDV expression compared with the other congeners. Based on the immunofluorescence findings, Western blot analysis was subsequently performed (Figure 4C,D and Figure S1), confirming that procyanidins A1 and B1 exerted more significant and dose-dependent inhibitory effects. Ribavirin served as a positive control drug (Figure S2).

**Figure 5:**
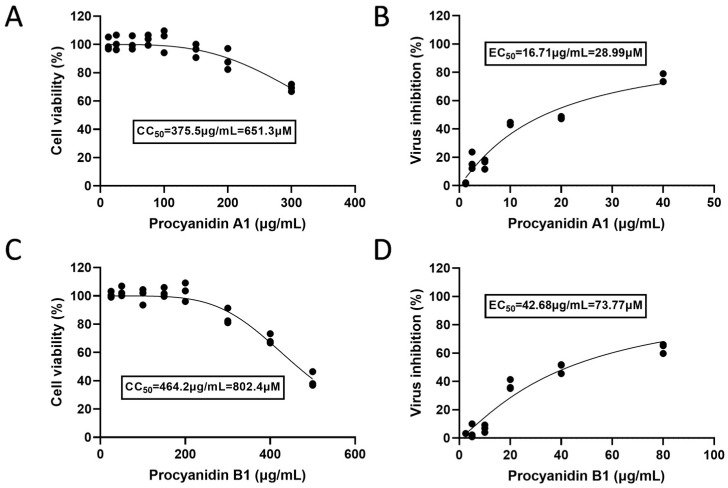
Drug selectivity index of procyanidins A1 and B1 (*n* = 3). (**A**) CC_50_ of procyanidin A1 in Vero cells. (**B**) EC_50_ of procyanidin A1 in Vero cells. (**C**) CC_50_ of procyanidin B1 in Vero cells. (**D**) EC_50_ of procyanidin B1 in Vero cells.

To verify the safety and efficacy of procyanidins A1 and B1, the CC_50_ and EC_50_ of the compounds were measured, and the drug selectivity index was calculated. The results showed that in Vero cells (Figure 5), the SI of procyanidin A1 was 22.4, and that of procyanidin B1 was 10.8. In addition, we also determined the CC_50_ and EC_50_ in IPEC-J2 cells (Figure S3), which showed that the SI of procyanidin A1 was 22.8, and that of procyanidin B1 was 8.0.

### 3.4. Procyanidins A1 and B1 Primarily Inhibit PEDV During the Adsorption, Internalization, and Replication Stages and Possess Direct Virucidal Activity

**Figure 6:**
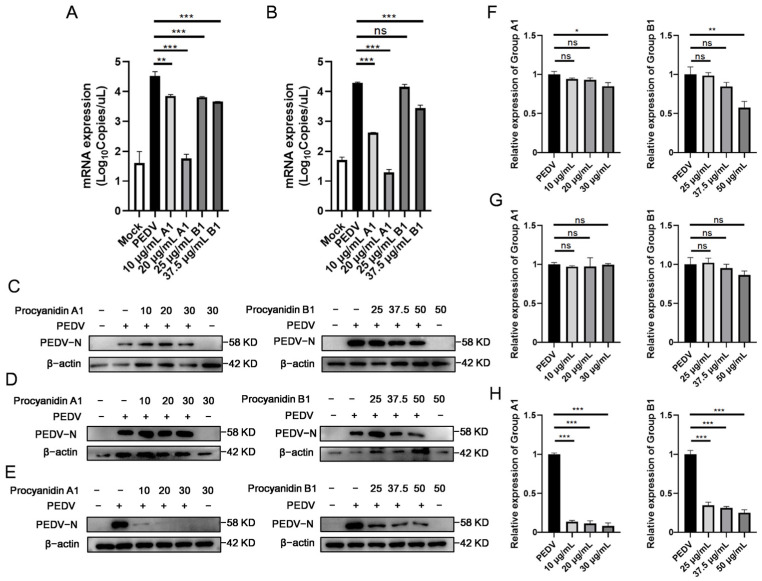
Inhibitory effects of procyanidins A1 and B1 on different stages of the PEDV life cycle (*n* = 3). (**A**) Viral Adsorption: Vero cells were precooled at 4 °C and then incubated with a mixture of procyanidins and PEDV (MOI = 0.5) for 1 h. The cells were then processed for RT-qPCR. (**B**) Viral internalization: Vero cells were precooled at 4 °C and infected with PEDV (MOI = 0.5) for 1 h. The inoculum was removed, and the cells were incubated with procyanidins for 2 h. The cells were then processed for RT-qPCR. (**C**) Viral replication: Vero cells were infected with PEDV (MOI = 0.5) at 37 °C for 1 h. The inoculum was removed, and the cells were treated with procyanidins for 2 h. Total protein was extracted for Western blot. (**D**) Viral release: Vero cells were infected with PEDV (MOI = 0.5) at 37 °C for 1 h. The inoculum was removed, and the cells were treated with procyanidins for 12 h. Total protein was extracted for Western blot. (**E**) Direct inactivation: PEDV (MOI = 0.5) was mixed with procyanidins, incubated at 37 °C for 1 h, washed with PBS, and then subjected to ultracentrifugation at 90,000× *g* at 4 °C to purify the virus. The viral particles were subsequently resuspended in culture medium and inoculated into Vero cells, and cell lysates were collected for Western blot analysis. (**F**–**H**) Densitometric analysis of protein bands was performed via ImageJ 1.54g software, corresponding to (**C**–**E**), respectively. ns: *p* > 0.05, *: *p* < 0.05, **: *p* < 0.01, ***: *p* < 0.001.

To elucidate the specific stage of the viral replication cycle targeted by procyanidins A1 and B1, we conducted time-of-addition assays. Drug treatments were administered at different time points during PEDV infection of Vero cells, followed by RNA or protein extraction for RT–qPCR and Western blot analyses, respectively. The RT–qPCR results demonstrated that procyanidins A1 and B1 significantly reduced viral mRNA expression levels during the viral adsorption and internalization stages (Figure 6A,B). The Western blot results indicated that procyanidins A1 and B1 effectively suppressed viral protein expression during the replication stage (Figure 6C). Additionally, these procyanidins had significant direct virucidal effects (Figure 6E). However, the results suggested that procyanidins A1 and B1 had negligible effects on the viral release stage (Figure 6D).

### 3.5. Procyanidins A1 and B1 Antagonize PEDV-Induced Alterations in Autophagy, Mitophagy, and Mitochondrial Dynamics Proteins

**Figure 7:**
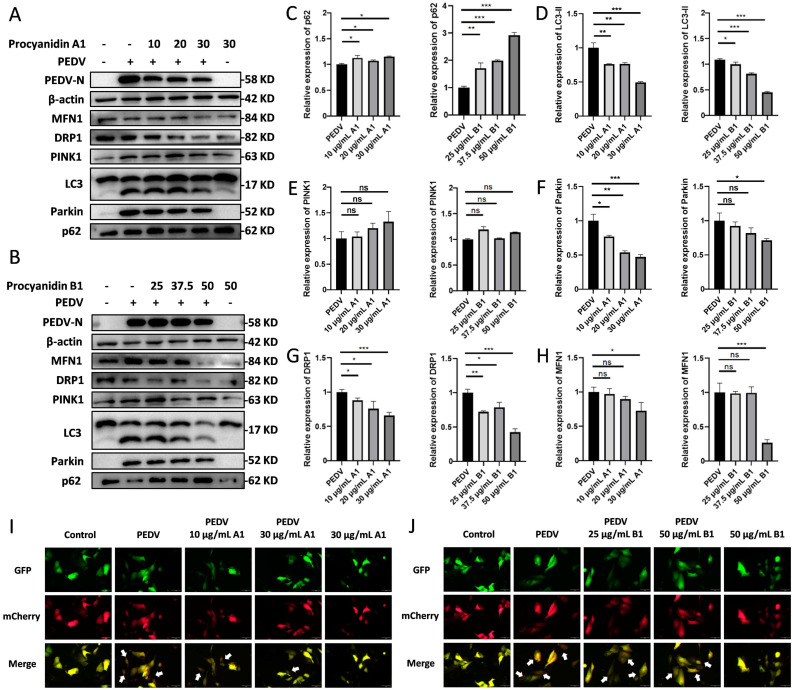
Procyanidins A1 and B1 antagonize the effects of PEDV on autophagy, mitochondrial dynamics, and mitophagy-related proteins (*n* = 3). (**A**,**B**) Vero cells were infected with PEDV (MOI = 0.5) for 2 h, followed by treatment with procyanidins A1 or B1. (**A**) Cells were treated with procyanidin A1 (10, 20, or 30 μg/mL) for 20 h, and total protein was extracted for Western blot. (**B**) Cells were treated with procyanidin B1 (25, 37.5, or 50 μg/mL) for 20 h, and total protein was extracted for Western blot. (**C**–**H**) Densitometric analysis of the p62 (**C**), LC3-II (**D**), PINK1 (**E**), Parkin (**F**), DRP1 (**G**), and MFN1 (**H**) protein bands was performed via ImageJ. (**I**,**J**) IPEC-J2 cells were transfected with the mCherry-GFP-LC3 plasmid, and the effects of procyanidins on autophagic flux during PEDV infection were evaluated via fluorescence microscopy (scale bar 10 μm). Arrows indicate representative yellow or red puncta of mCherry-GFP-LC3, reflecting autophagosomes or autolysosomes. (**I**) Procyanidin A1 treatment group. (**J**) Procyanidin B1 treatment group. ns: *p* > 0.05, *: *p* < 0.05, **: *p* < 0.01, ***: *p* < 0.001.

Given that PEDV infection perturbs autophagic flux and disrupts mitochondrial homeostasis, we investigated whether the antiviral effects of procyanidins involve the modulation of these processes. We analyzed the protein expression of LC3-II and p62 in PEDV-infected Vero cells treated with procyanidins A1 and B1 via Western blot (Figure 7C,D). The results indicated that the expression of p62 was restored in the procyanidin-treated groups while LC3-II expression was decreased, suggesting that treatment reversed the PEDV-induced autophagy. Meanwhile, IPEC-J2 cells were transfected with the mCherry-GFP-LC3 plasmid, and were visualized using fluorescence microscopy (Figure 7I,J). The results show that PEDV infection induces extensive punctate mCherry-GFP-LC3 signals in IPEC-J2 cells, whereas procyanidin treatment restores a uniform yellow fluorescence with only sparse scattered yellow or red puncta, consistent with the Western blot data. Furthermore, the uniform yellow fluorescence observed in procyanidin-treated uninfected cells indicates that procyanidins do not affect basal autophagy in normal cells but act specifically under infection conditions.

Furthermore, since PEDV infection downregulates MFN1 and upregulates DRP1 to promote mitochondrial fission and trigger mitophagy, we sought to determine whether procyanidins could modulate this pathway. We examined the protein levels of MFN1, DRP1, PINK1, and Parkin. Compared with the PEDV-infected group, the groups treated with procyanidins A1 and B1 presented significantly lower expression levels of Parkin (Figure 7F) and DRP1 (Figure 7G). These findings suggest that procyanidins effectively attenuate the abnormal upregulation of DRP1 and Parkin induced by PEDV, thereby antagonizing the impact of viruses on mitochondrial dynamics and mitophagy.

### 3.6. Procyanidins A1 and B1 Show Potential Binding Affinity to the MFN1, DRP1, PINK1, and Parkin Proteins

**Figure 8:**
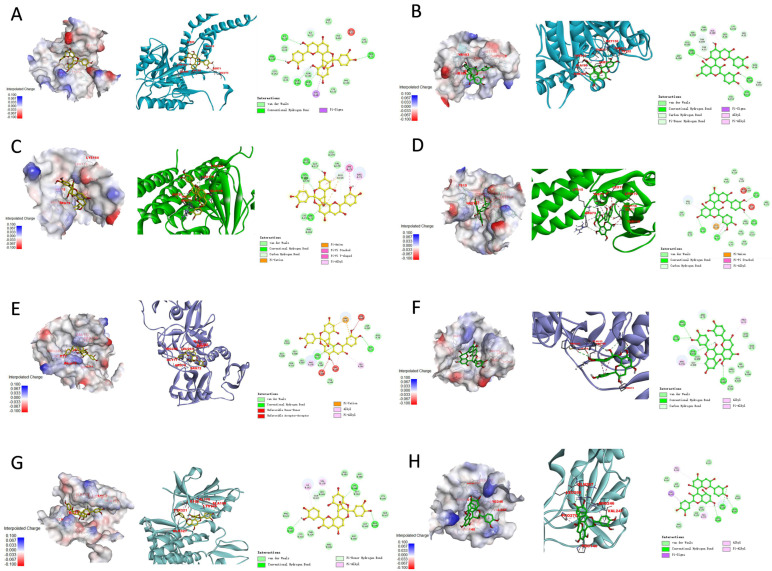
Molecular docking simulations of procyanidins A1 and B1 with proteins related to mitophagy and mitochondrial membrane dynamics. Panels (**A**–**H**) Display the molecular docking results (binding poses) and 2D diagrams illustrating hydrogen bonds and detailed intermolecular interactions between the procyanidins and target proteins. (**A**) Procyanidin A1 with DRP1. (**B**) Procyanidin B1 with DRP1. (**C**) Procyanidin A1 with MFN1. (**D**) Procyanidin B1 with MFN1. (**E**) Procyanidin A1 with Parkin. (**F**) Procyanidin B1 with Parkin. (**G**) Procyanidin A1 with PINK1. (**H**) Procyanidin B1 with PINK1.

To further investigate the molecular mechanisms underlying the potent antiviral efficacy of procyanidins, we performed molecular docking analyses via QuickVina-W to predict the binding interactions of these compounds with mitochondrial dynamics proteins (MFN1 and DRP1) and mitophagy-related proteins (PINK1 and Parkin) (Figure 8 and Figure S4). The docking results showed that procyanidins A1 and B1 have potential binding affinities for MFN1, DRP1, PINK1, and Parkin. Notably, procyanidin A1 achieved a high docking score of −10.8 kcal/mol with Parkin, which was significantly higher than that of the other configurations (Table 3 and Table S1). These findings suggest that procyanidin A1 may exert its antiviral effects by targeting Parkin and participating in the regulation of cellular pathways.

## 4. Discussion

Our previous studies demonstrated the significant inhibitory capacity of procyanidins against PEDV infection. The present study aimed to elucidate the interaction mechanisms between procyanidins and the host, thereby revealing potential therapeutic targets for PEDV.

During the long-term coevolution of viruses and their hosts, viruses have evolved various strategies to hijack autophagy to facilitate their own replication. For example, infections with influenza A virus (IAV) and porcine reproductive and respiratory syndrome virus (PRRSV) trigger autophagosome formation, leading to the accumulation of viral RNA and proteins to increase replication [5]. PEDV infection results in a similar phenomenon. We observed that following PEDV infection, the expression of the autophagy-related proteins LC3-II increased and p62 decreased to varying degrees. These findings suggest that PEDV infection may either excessively activate autophagic flux or hijack autophagy, thereby facilitating viral replication. Our mCherry-GFP-LC3 plasmid transfection experiment also confirms this conclusion. Concurrently, we noted downregulation of the mitochondrial fusion protein MFN1 and upregulation of the fission protein DRP1. Under normal conditions, mitochondria regulate their morphology through a balance between fission and fusion—known as mitochondrial dynamics—to adapt to the cellular environment [18]. This process involves ultrastructural remodeling and affects not only cellular metabolism but also complex signaling events, such as cell senescence and death. Specifically, DRP1 promotes mitochondrial fission through various modifications, subsequently activating mitophagy [17], while the mitophagy-related protein PINK1 also plays an important role in the degradation of MFN1 [19]. Indeed, during PEDV infection, we observed increased expression of the core mitophagy proteins PINK1 and Parkin, indicating disruption of mitochondrial homeostasis. By knocking down or overexpressing the mitochondrial membrane dynamics proteins DRP1 and MFN1, we indeed observed a regulatory effect on the expression of PINK1 and Parkin proteins. Interestingly, the regulation of PINK1 and Parkin proteins by MFN1 exhibits an asymmetric effect, which may be attributable to either the existence of a compensatory regulatory network parallel to the PINK1/Parkin pathway, or the balanced regulation of MFN1 stability and function by multiple post-translational modifications, creating a “threshold effect” [20,21].

Our time-of-addition assays demonstrated that procyanidins A1 and B1 exert their most potent antiviral effects during the early stages of infection, specifically via direct virucidal inactivation and blockade of viral adsorption and internalization. This finding suggests that the primary antiviral barrier is established extracellularly or at the cell membrane. However, for virions that successfully bypass this initial defense and enter the host cell, our data reveal a critical secondary line of defense involving the restoration of mitochondrial homeostasis [22]. PEDV infection typically triggers excessive mitochondrial fission and mitophagy to facilitate its replication, likely by hijacking the mitochondrial membrane and providing energy [8]. Meanwhile, our previous studies have shown that procyanidins can restore the mitochondrial morphology disrupted by PEDV [11]. In this study, we found that procyanidins A1 and B1 effectively counteract this process by reversing the virus-induced upregulation of DRP1 and Parkin. This modulation presumably deprives the virus of the specific mitochondrial environment required for efficient propagation [23]. Therefore, we propose that procyanidins A1 and B1 may exert their effects through a synergistic “dual-action” mechanism: by inactivating viral particles or blocking their adsorption and internalization to prevent viral entry, while simultaneously modulating host mitochondrial proteins to suppress the replication of viruses that have already entered the cells.

Procyanidins are chiral molecules. To further validate the possibility that procyanidins affect mitochondria through this pathway, we performed molecular docking simulations of procyanidins A1 and B1 with MFN1, DRP1, PINK1, and Parkin. The results revealed potentially high binding affinities between both procyanidins and these proteins. Notably, the docking score of procyanidin A1 with Parkin reached –10.8 kcal/mol, representing the highest potential binding energy among the four proteins. Notably, procyanidin A1 exerted a more significant effect than procyanidin B1 in reversing the PEDV-induced upregulation of Parkin protein expression. Our supplementary molecular docking simulations also revealed that procyanidin A1 exhibits a significantly higher binding affinity for Parkin than the other configurations, suggesting the possibility of this intracellular mechanism, and indicating that it may physically interact with proteins through hydrophobic forces, hydrogen bonds, and other interactions to prevent aberrant activation of the proteins. This may explain the superior efficacy of A1 during the viral adsorption, internalization, and replication stages. Our study revealed a distinct structure–activity relationship (SAR) among the tested procyanidins. Interestingly, procyanidins A1 and B1 exhibited significantly stronger antiviral activities than A2 and B2 did. While previous studies focused on the differences in rigidity between A-type (double linkage) and B-type (single linkage) procyanidins [24], our data suggest that the linkage type is not the sole determinant. Structural comparison revealed that procyanidins A1 and B1 share a terminal (+)-catechin unit, whereas A2 and B2 possess a terminal (−)-epicatechin unit. These findings suggest that the stereochemistry of the terminal flavan-3-ol unit may play a key role in anti-PEDV activity [25]. The trans-configuration of the 3-hydroxyl group in the terminal catechin may facilitate more stable hydrogen bonding or hydrophobic interactions with viral proteins or the host mitochondrial receptor Parkin [26,27], as supported by our docking scores. In contrast, the axial orientation of the cis-configuration in the terminal epicatechin may induce steric hindrance upon entering the hydrophobic pocket of Parkin, forcing a shift in binding pose and disrupting the specific “functional conformational induction” [28,29]. These findings provide novel chemical insights for the design of anti-PEDV agents.

In conclusion, our results demonstrate that procyanidins A1 and B1 exert potent inhibitory effects during the adsorption, internalization, and replication stages of PEDV CV777 infection, and also exhibit significant direct virucidal activity. Moreover, procyanidins A1 and B1 restore the PEDV CV777-induced alterations in autophagy-related proteins, mitophagy-related proteins, and mitochondrial membrane dynamics proteins. Molecular docking predictions further suggest that procyanidins have potential binding affinity for the Parkin protein, with stereochemical differences among them. This study reveals potential therapeutic targets for PEDV and provides new insights for targeted antiviral therapies. However, limitations remain, such as the need to fully elucidate how procyanidins and mitophagy specifically influences the viral life cycle and whether this connects to our previous findings regarding the ability of procyanidins to rescue virus-induced interferon suppression. Furthermore, direct interactions between procyanidins and proteins such as DRP1/Parkin, as well as upstream regulatory signals and functional rescue experiments, remain to be verified. Therefore, our future work will focus on exploring the crosstalk among host cell metabolism, mitophagy mechanisms, and virus-related immune responses, combined with target validation such as CETSA and gene knockout/overexpression models, in order to further clarify the molecular mechanisms of procyanidin-mediated PEDV inhibition and aid in the development of anti-PEDV drugs.

## Figures and Tables

**Table 1:** Primers used for plasmids construction.

Title 1	Primer Sequences (5′-BamHI, 3′-XhoI)
MFN1-Forward	CGCGGATCCATGGCAGAAACTGCTTCTCCACTGA
MFN1-Reverse	CCGCTCGAGAGATTCTTCATTGCTTGAATG
DNM1L-Forward	CGCGGATCCATGGAGGCGCTAATTCCTGTCATAAACAAG
DNM1L-Reverse	CCGCTCGAGTCCACCAAAGATGAGTCTCCCGGATTT

**Table 2:** Primer information used in the study.

Gene Name	Primer Sequences (5′-3′)
PEDV-N-F	TTGTTGAACCTAACACACCTCC
PEDV-N-R	ACAGCAGCCACCAGATCATC

**Table 3:** Vina docking scores for proteins in the study.

Protein Name	PDB ID	Vina Docking Score with Procyanidin A1	Vina Docking Score with Procyanidin B1
DRP1	3W6O	−8.5	−7.7
MFN1	5GOF	−8.9	−8.7
PINK1	9EII	−8.9	−7.8
Parkin	5C1Z	−10.8	−8.9

## Data Availability

The original contributions presented in this study are included in the article. Further inquiries can be directed to the corresponding authors.

## Supplementary Materials

The following supporting information can be downloaded at: https://www.mdpi.com/article/10.3390/v18070758/s1, Figure S1: Inhibitory effects of different procyanidin configurations on PEDV infection; Figure S2: Ribavirin inhibits the PEDV infection; Figure S3: Drug selectivity index of procyanidins A1 and B1 in IPEC-J2 cells; Figure S4: Molecular docking simulations of procyanidins A2 and B2 with Parkin; Table S1: Vina docking scores for procyanidin A2 and B2 with Parkin.
