# Supplementary material for: Procyanidins A1 and B1 Suppress PEDV CV777 by Modulating Mitophagy
Source: Viruses. 2026 Jul 10;18(7):758. doi: 10.3390/v18070758 (PMC13431556; doi:10.3390/v18070758)
Supplement: Supplementary file 1 [file viruses-18-00758-s001.zip › viruses-4337192-supplementary.pdf]

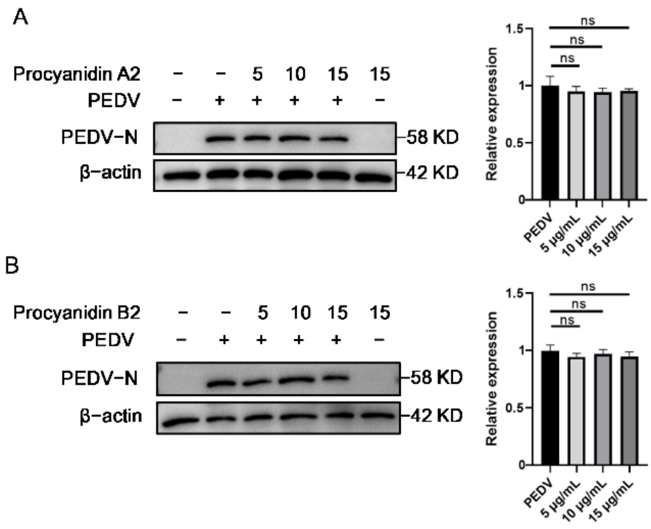

Figure S1. Inhibitory effects of different procyanidin configurations on PEDV infection (n = 3). (A–B) Vero cells were infected with PEDV (MOI = 0.5) for 2 h and subsequently treated with the indicated concentrations of procyanidins for 20 h. Total protein was extracted for Western blot analysis. (A) Procyanidin A2 at 5, 10 and 15 μg/mL. (B) Procyanidin B2 at 5, 10, and 15 μg/mL. *$p < 0.05$, **$p < 0.01$, and ***$p < 0.001$

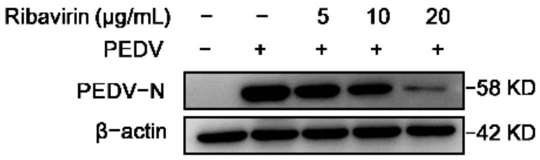

Figure S2. Ribavirin inhibits the PEDV infection.

PEDV and β-actin were analyzed by western blotting for indicated protein levels in the Vero cells.

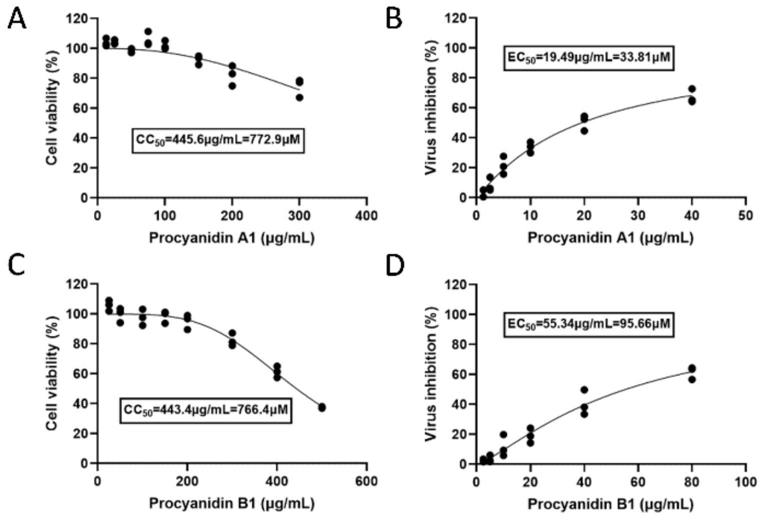

Figure S3. Drug selectivity index of procyanidins A1 and B1 in IPEC-J2 cells. **(A)** CC_{50} of procyanidin A1 in IPEC-J2 cells. **(B)** EC_{50} of procyanidin A1 in IPEC-J2 cells. **(C)** CC_{50} of procyanidin B1 in IPEC-J2 cells. **(D)** EC_{50} of procyanidin B1 in IPEC-J2 cells.

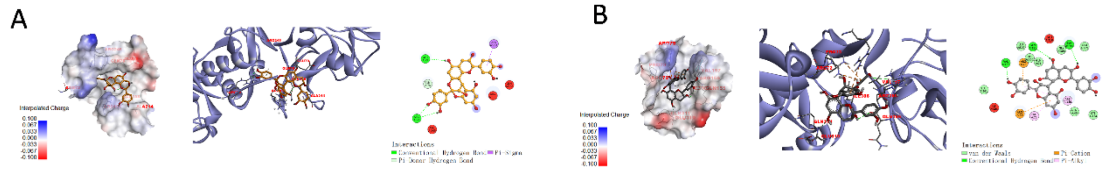

Figure S4. Molecular docking simulations of procyanidins A2 and B2 with Parkin. **(A–B)** Display the molecular docking results (binding poses) and 2D diagrams illustrating hydrogen bonds and detailed intermolecular interactions between the procyanidins and target proteins. **(A)** Procyanidin A2 with Parkin. **(B)** Procyanidin B2 with Parkin.

Table S1. Vina docking scores for procyanidin A2 and B2 with Parkin.

Protein Name	PBD ID	Vina docking score with procyanidin A2	Vina docking score with procyanidin B2
Parkin	5C1Z	-9.7	-9.0
